# Feasibility of assessing the needs of stroke patients after six months using the GM-SAT

**DOI:** 10.1177/0269215512457403

**Published:** 2013-03

**Authors:** Katy Rothwell, Ruth Boaden, David Bamford, Pippa J Tyrrell

**Affiliations:** 1 NIHR Collaboration for Leadership in Applied Health Research and Care (CLAHRC) for Greater Manchester, Salford Royal NHS Foundation Trust, UK; 2 Manchester Business School, University of Manchester, UK; 3 Business School, University of Huddersfield, UK; 4 University of Manchester and Manchester Academic Health Sciences Centre, Salford Royal NHS Foundation Trust, UK

**Keywords:** Needs, assessment, stroke, long-term, GM-SAT

## Abstract

**Objective:**

To investigate the feasibility of administering the Greater Manchester Stroke Assessment Tool (GM-SAT), a structured evidence-based needs assessment tool, in a community setting and its acceptability to stroke patients and their carers.

**Setting:**

Community stroke services.

**Subjects:**

One hundred and thirty-seven stroke patients at six months post hospital discharge with no communication or cognitive difficulties residing in their own homes.

**Intervention:**

Patients’ needs were assessed by information, advice and support (IAS) coordinators from the UK Stroke Association using the GM-SAT.

**Main measures:**

Number and nature of unmet needs identified and actions required to address these; patient/carer feedback; and IAS coordinator feedback.

**Results:**

The mean number of unmet needs identified was 3 (min 0, max 14; SD 2.5). The most frequently identified unmet needs related to fatigue (34.3%), memory, concentration and attention (25.5%), secondary prevention non-lifestyle (21.9%) and depression (19.0%). It was found that 50.4% of unmet needs could be addressed through the provision of information and advice. Patients/carers found the assessment process valuable and IAS coordinators found the GM-SAT easy to use.

**Conclusions:**

Results demonstrate that the GM-SAT is feasible to administer in the community using IAS coordinators and is acceptable to patients and their carers, as well as staff undertaking the assessments. Further research is needed to determine whether the application of the GM-SAT at six months improves outcomes for patients.

## Introduction

Stroke is increasingly considered a long-term condition that requires appropriate clinical management to optimize patient outcomes and quality of life.^1^ There is recognition that people who have had a stroke, and their carers, need to be supported in the months and years following the event. During this time they experience significant changes, both positive and negative, in their health, physical, social and emotional care needs.^2–4^ For many, the full impact of a stroke is only realized following discharge from hospital or community rehabilitation when they are left to adjust to the full impact of the stroke on their life at home or in a care home.^4–6^ Unfortunately, at this stage, patients and carers may report a sense of ‘abandonment’ and have considerable difficulty accessing the services and support they require.^4–6^

The English National Stroke Strategy,^7^ in common with other national policies,^8–10^ requires that patients and their carers receive regular reviews of their health and social care needs after stroke, including a review specifically six months following discharge from hospital. Such reviews are designed to ensure that patients feel supported in the long term and to provide access to further specialist review, advice, information, support and rehabilitation where appropriate. There is, however, currently no single systematic structured assessment tool in widespread use in England.^11^

Attempts to develop outcome evidence for this type of review are both limited and inconclusive, and recommendations in various national stroke strategies are ‘not supported by any trial evidence’.^12^ A recent trial found a review at six months to not be associated with any clinically significant benefit at 12 months.^13^ However, the requirement to identify and address the needs of patients after stroke remains an international priority. The lack of trial evidence while there is a clear service need is an example of the tensions arising from different epistemologies of improvement which may result in the view that, in certain situations, including this one, it ‘can be morally justifiable to “just do it and learn as you go”’.^14^

The Greater Manchester Stroke Assessment Tool (GM-SAT) is a descriptive post-stroke assessment tool developed to provide a structured, systematic needs assessment six months post hospital discharge. We investigated the feasibility of administering the GM-SAT in a community setting and its acceptability to patients and their carers.

## Methods

The study was based in ten community stroke services in England which serve a collective population of approximately 2.25 million.

Fifteen experienced information, advice and support (IAS) coordinators from the UK Stroke Association were recruited to undertake six-month assessments using the GM-SAT as an extension to their usual work. Coordinators were support workers and were not required to have formal clinical qualifications. All coordinators received standardized training in the use of the GM-SAT prior to completing any assessments.

Patients were identified from the coordinators’ existing caseloads and were deemed suitable for inclusion in the study if they had a clinically confirmed stroke (excluding subarachnoid haemorrhage), were over 18 years of age and, in line with the criteria for six-month reviews provided by the NHS Stroke Improvement Programme,^15^ had been discharged from hospital 5–7 months previously. Participants were excluded if they resided in nursing or residential care or were known to have communication or significant cognitive problems as the GM-SAT was not considered by the authors as fit for purpose in these populations at this stage of its development.

Coordinators contacted eligible patients by telephone. During this contact they explained the aims of the study and sought verbal consent to participate. Formal ethical approval was not required for the study since it was classified by NHS governance procedures as service evaluation.

For patients agreeing to participate, a structured assessment using the GM-SAT was completed by a coordinator in the patient’s home. Patients were either assessed alone or, where an informal carer was present and the patient consented, as a patient–carer ‘unit’.

The GM-SAT covers 34 patient- and one carer-related common, long-term, post-stroke problem areas. For each area, the GM-SAT provides assessment questions and a simple algorithm that directs assessors to the most appropriate evidence-based management option for any given unmet need (http://clahrc-gm.nihr.ac.uk/resources/gm-sat/5/).

During the assessment, coordinators worked systematically through the questions in the GM-SAT, recording any unmet needs identified. An unmet need was defined as ‘a problem that was not being addressed or one that was being addressed, but insufficiently’. The coordinator recorded any actions that had been, or were to be, taken to address the unmet needs identified. Actions included making referrals to specialist services, signposting patients to community services and the provision of information and advice. Problems not amenable to resolution, such as fatigue, were still classed as unmet need where self-management information and advice was required.

Following each assessment, coordinators completed a summary report, documenting any unmet needs identified and the actions that had been agreed with the patient and, where appropriate, his or her carer. Coordinators also completed any actions required of themselves, such as making onward referrals to other services.

Summary reports were anonymized and reviewed by project leads who, using a data extraction form, recorded the number and nature of unmet needs identified at each assessment and the actions taken.

After each assessment, patients and their carers were given a structured feedback questionnaire. This comprised open- and close-ended questions to ascertain patients’ and carers’ views of the assessment. While the majority of the questionnaire was purposely constructed for use in the study, an adapted version of the consultation quality index (CQI)^16^ was used, a validated tool ordinarily used in general practice to measure the holism and patient-centredness of a patient consultation.

A structured feedback questionnaire was completed by coordinators after each assessment. This contained open- and closed-ended questions designed to elicit their views on the usefulness of the GM-SAT and the assessment process and to identify any difficulties encountered. In addition, coordinators recorded the direct (i.e. time with the patient) and indirect (i.e. time spent arranging the assessment and completing actions) time required to complete the assessment.

## Results

One hundred and thirty-seven patients were assessed, with a mean age of 72.6 years (min 40, max 93). 44.5% (*n* = 61) were female and, in 47.4% (*n* = 65) of cases, an informal carer was present for the duration of the assessment. No patients approached refused to participate in the study.

A total of 464 unmet needs were identified during the study, with 8.0% (*n* = 11) of patients having no unmet needs. Patients presented with a mean of three unmet needs (median 2; SD 2.5), ranging from 0 to 14 unmet needs per patient. Unmet needs were identified in 34 of the 35 areas covered by the GM-SAT.

Table 1 details the nature and frequency of unmet needs identified. The two areas not covered by the GM-SAT in which unmet needs were identified were foot care (*n* = 2; 1.5%) and will-making (*n* = 1; 0.7%). No patients in this sample identified post-stroke seizures as a problem.

**Table 1.** Frequencies of unmet needs identified using the GM-SAT

	(*n* = 137)
Medication management	4 (2.9%)
Medication compliance	18 (13.1%)
Secondary prevention non-lifestyle	30 (21.9%)
Alcohol	7 (5.1%)
Diet	9 (6.6%)
Smoking	10 (7.3%)
Exercise	18 (13.1%)
Vision	8 (5.8%)
Hearing	8 (5.8%)
Communication	13 (9.5%)
Swallowing	7 (5.1%)
Nutrition	6 (4.4%)
Weight management	8 (5.8%)
Pain	12 (8.8%)
Headaches/migraines	9 (6.6%)
Seizures	0 (0.0%)
Continence	13 (9.5%)
Activities of daily living	13 (9.5%)
Mobility	9 (6.6%)
Falls	10 (7.3%)
Depression	26 (19.0%)
Anxiety	20 (14.6%)
Emotionalism	4 (2.9%)
Personality changes	16 (11.7%)
Sexual health	4 (2.9%)
Fatigue	47 (34.3%)
Sleep pattern	11 (8.0%)
Memory, concentration and attention	35 (25.5%)
Driving	13 (9.5%)
Transport and travel	7 (5.1%)
Activities and hobbies	11 (8.0%)
Employment	9 (6.6%)
Benefits and finances	25 (18.2%)
House and home	10 (7.3%)
Carer/supporter needs	11 (8.0%)
Other	3 (2.2%)
TOTAL	464

To address the unmet needs identified, 464 actions were recorded. The provision of verbal or written information and advice, accounted for half of all actions undertaken (*n* = 234; 50.4%). Ninety-two (19.8%) unmet needs were addressed by signposting patients and their carers to community services, such as those providing benefits advice and exercise opportunities. In response to 98 (21.1%) unmet needs identified, patients were advised to make an appointment with their primary care team.

Forty (8.6%) unmet needs required the patient to be referred to other services. However, as on several occasions patients were referred to the same service for more than one unmet need, 37 actual referrals were made. Table 2 details the frequency and nature of the referrals made.

**Table 2.** Frequencies of referrals made to services

	(*n* = 137)
Audiology	3 (2.2%)
Communication support service	3 (2.2%)
Continence advisory service	5 (3.6%)
Counselling service	2 (1.5%)
Dietetics	1 (0.7%)
Falls clinic	2 (1.5%)
Falls prevention service	1 (0.7%)
Occupational therapy	4 (2.9%)
Physiotherapy	3 (2.2%)
Psychology	2 (1.5%)
Social services	5 (3.6%)
Speech and language therapy	5 (3.6%)
Visual impairment service	1 (0.7%)
TOTAL	37

One hundred and one patient and carer feedback questionnaires were returned, giving an overall response rate of 73.7%. All responders rated the review they had received as good or better, 30.7% (*n* = 31) rating it very good and 48.5% (*n* = 49) excellent. Table 3 shows further responses provided to the questionnaire.

**Table 3.** Patient and carer feedback

	Strongly agree	Agree	Neither agree nor disagree	Disagree	Strongly disagree
	*n* (%)	*n* (%)	*n* (%)	*n* (%)	*n* (%)
I appreciated the opportunity to discuss my needs (*n* = 101)	57 (56.4%)	40 (39.6%)	4 (4.0%)	0 (0.0%)	0 (0.0%)
I found it easy to talk about my needs and concerns (*n* = 101)	52 (51.5%)	47 (46.5%)	2 (2.0%)	0 (0.0%)	0 (0.0%)
I felt comfortable answering all the questions asked (*n* = 100)	51 (51.0%)	46 (46.0%)	2 (2.0%)	0 (0.0%)	1 (1.0%)
All my needs and concerns were addressed (*n* = 101)	46 (45.5%)	49 (48.5%)	4 (4.0%)	2 (2.0%)	0 (0.0%)
My coordinator knew how to help me (*n* = 99)	58 (58.6%)	39 (39.4%)	2 (2.0%)	0 (0.0%)	0 (0.0%)
I was given all the information and advice I needed (*n* = 99)	49 (49.5%)	46 (46.5%)	4 (4.0%)	0 (0.0%)	0 (0.0%)
Information and advice was given in a way that was easy to understand (*n* = 100)	58 (58.0%)	41 (41.0%)	0 (0.0%)	1 (1.0%)	0 (0.0%)
My carer/relative/friend was sufficiently involved (if applicable) (*n* = 75)	31 (41.3%)	39 (52.0%)	5 (6.7%)	0 (0.0%)	0 (0.0%)
The review was valuable (*n* = 101)	47 (46.5%)	45 (44.6%)	7 (6.9%)	2 (2.0%)	0 (0.0%)

Patients and carers also used the feedback questionnaire to express how the assessment made them feel supported. They indicated that they had found the opportunity to talk about their needs and to work with the coordinator to address these highly beneficial. In addition, patients voiced how the structured and comprehensive nature of the assessment gave them an opportunity to discuss issues they would not have otherwise raised, such as those relating to sexual health.

One hundred and thirty-two questionnaires were completed by the coordinators involved in the study. Coordinator feedback is summarized in Table 4.

**Table 4.** Coordinator feedback

	Strongly agree	Agree	Neither agree nor disagree	Disagree	Strongly disagree
	*n* (%)	*n* (%)	*n* (%)	*n* (%)	*n* (%)
I felt comfortable undertaking the assessment (*n* = 132)	87 (65.9%)	45 (34.1%)	0 (0.0%)	0 (0.0%)	0 (0.0%)
I had the skills required to complete the assessment (*n* = 132)	88 (66.7%)	42 (31.8%)	1 (0.8%)	1 (0.8%)	0 (0.0%)
Conversation focused on the needs and concerns expressed by the patient (*n* = 132)	90 (68.2%)	42 (31.8%)	0 (0.0%)	0 (0.0%)	0 (0.0%)
I knew how to address the needs and concerns expressed by the patient (*n* = 132)	87 (65.9%)	45 (34.1%)	0 (0.0%)	0 (0.0%)	0 (0.0%)
The assessment tool was easy to use (*n* = 123)	85 (69.1%)	33 (26.8%)	5 (4.1%)	0 (0.0%)	0 (0.0%)
The assessment tool helped me to discuss topics with the patient that I would not have otherwise discussed (*n* = 122)	34 (27.9%)	36 (29.5%)	23 (18.9%)	19 (15.6%)	10 (8.2%)
The assessment tool helped me to explore sensitive issues with the patient (*n* = 122)	39 (32.0%)	48 (39.3%)	26 (21.3%)	9 (7.4%)	0 (0.0%)
I was able to give the patient my full attention during the assessment (*n* = 132)	96 (72.7%)	35 (26.5%)	0 (0.0%)	1 (0.8%)	0 (0.0%)
I felt the service user benefited from having the assessment (*n* = 131)	76 (58.0%)	48 (36.6%)	7 (5.3%)	0 (0.0%)	0 (0.0%)
I would like to offer this assessment to all my patients (*n* = 131)	94 (71.8%)	36 (27.5%)	1 (0.8%)	0 (0.0%)	0 (0.0%)

The assessment took a mean of 74 minutes to complete (min 20, max 195; SD 31.6). Only 3.0% (*n* = 3) of patients considered this to be too long. A mean of 33 minutes (min 10, max 125; SD 22.0) of indirect time was required to arrange an appointment for the assessment and to complete associated paperwork.

## Discussion

Our study has shown that the GM-SAT is feasible to administer in the community and is acceptable to patients and their carers, as well as to staff undertaking the assessments. We found that, six months post hospital discharge, patients and their carers had a mean of three unmet needs (min 0, max 14), comparable to the number identified by other studies.^17,18^ The content of the GM-SAT appears to be reasonably comprehensive, with unmet needs being identified in all but one of the 35 areas covered. The areas in which unmet needs were identified show considerable overlap with those identified in previous studies.^13,17^ Our results are also consistent with qualitative and quantitative studies that have shown that long-term stroke problems are diverse and are frequently primarily psychosocial in nature.^2,3,6^

The study demonstrates that half of all unmet needs identified (50.4%, *n* = 234) can be addressed through the provision of information and advice at the point of assessment. This should provide reassurance to service providers who may have been concerned that the implementation of post-stroke needs assessments would lead to community health, rehabilitation and social services being overwhelmed with referrals for further support and intervention. Needs assessments, such as those demonstrated here however, could be effective in identifying gaps in national and local service provision and could be useful in supporting service development.

All patients reported that they had found the assessment beneficial and that the experience of participating in an assessment made them feel supported. A significant number expressed that they felt comfortable and at ease during their assessment, in an atmosphere which offered an open, honest forum in which they could talk candidly about their needs. This may be because the assessment took place at home and that the patient and carer already had an established relationship with the coordinator administering the assessment. Patients typically reported that it was good to be ‘able to discuss things with someone who understands how stroke affects and changes a person and could help and advise on all these points’. Although patients found the assessment valuable, it would be difficult to quantify this in a randomized controlled trial as much of the value may have been simply in being given time to reflect on progress following stroke and being ‘allowed’ to comment on non-medical or rehabilitation issues such as fatigue and sexual function.

The structured assessment took coordinators an average of 74 minutes of direct time and 33 minutes of indirect time to complete. This allows for some estimation of the staff resources that would be required to implement post-stroke needs assessments. However, as the coordinators were new to the assessment, it would appear reasonable to assume that, with practice, time taken may reduce. The significant variation observed in the time required to undertake the assessment is likely to reflect the varying number and complexity of unmet needs with which each patient presented. The IAS coordinators found the GM-SAT easy to use and said that it helped them to explore sensitive issues with the patient. This is important as in the absence of specific enquiry, patients and their carers often fail to communicate their concerns to professionals, particularly those relating to psychosocial issues.^19–23^

There is currently no consensus of how or by whom an unmet need should be defined, although the importance of its assessment in clinical practice is undisputed.^24^ The assessment of unmet need in this study was primarily subjective, with an explicit focus on patient-defined unmet needs. Depending on individual definitions of unmet need, this may be considered a limitation of our study as needs perceived as unmet by a patient may be representative of a lack of specific evidence-based interventions available to ameliorate a given problem rather than an area that is not being sufficiently addressed by existing services. This could contribute to an overestimate of unmet need. However, it is possible that this was, to some extent, circumvented in the study by the coordinators who, although not specialist clinicians, could enquire further about any patient-identified unmet need and assist the patient in determining whether an identified need was a problem that could be further addressed.

In addition, although participants were aware that the assessment being conducted was in relation to their needs post stroke, it is possible that not all of the unmet needs reported were directly attributable to the stroke but could have been those present before the stroke or due to other comorbidities or circumstances. The applicability of the findings of this study across the total stroke population may also be questioned due to the highly selected sample of patients involved, not least because the patients involved were those who were already in contact with their assessors. It is possible that patients excluded from the study, such as those with communication and cognitive difficulties, may present with greater levels and different types of unmet needs six months post stroke. This is an area that we believe warrants further study.

While acknowledging the limitations of the current study, the findings indicate that the GM-SAT is a potentially useful tool for the identification of long-term post-stroke needs. Further research is needed to evaluate the applicability of the findings of this study in the wider stroke population and to assess the impact of six month post-discharge needs assessments employing the GM-SAT on clinical outcomes and quality of life.

**Clinical messages**

- The GM-SAT is feasible for use in a community setting and is acceptable to patients and carers, as well as staff undertaking the assessments.
- Problems concerning patients at six months post stroke are mainly psychosocial in nature and can be primarily addressed through the provision of information and advice.
